# Awareness and use of evidence-based medicine information among patients in Croatia: a nation-wide cross-sectional study

**DOI:** 10.3325/cmj.2017.58.300

**Published:** 2017-08

**Authors:** Danijel Nejašmić, Ivana Miošić, Davorka Vrdoljak, Snježana Permozer Hajdarović, Marion Tomičić, Rudika Gmajnić, Ines Diminić Lisica, Jelena Sironić, Hreljanović, Vlatka Pleh, Venija Cerovečki, Anita Tomljenović, Sanja Bekić, Minka Jerčić, Karla Tuđa, Livia Puljak

**Affiliations:** 1 Department of Medical Physics and Biophysics, University of Split School of Medicine, Split, Croatia; 2 Cochrane Croatia, University of Split School of Medicine, Split, Croatia; 3 Department of Family Medicine, University of Split School of Medicine, Split, Croatia; 4 Family Medicine Practice, Kotoriba, Croatia; 5 Family Medicine Practice, Osijek, Croatia; 6 Family Medicine Practice, Vežica, Rijeka, Croatia; 7 Family Medicine Practice, Viškovo, Rijeka, Croatia; 8 Family Medicine Practice, Čakovec, Croatia; 9 Department of Family Medicine, Andrija Štampar School of Public Health, University of Zagreb, School of Medicine, Zagreb, Croatia; 10 Health center Zagreb-Centar, Family Medicine Practice, Kupinečki Kraljevec, Croatia; 11 Family Medicine Practice, Višnjevac, Croatia; 12 Family Medicine Practice, Donji Muć, Croatia

## Abstract

**Aim:**

To determine the use of evidence-based medicine (EBM) information and the level of awareness and knowledge of EBM among patients in Croatia.

**Methods:**

A cross-sectional study was conducted among 987 patients in 10 family medicine practices in Croatia. Patients from both urban (n = 496) and rural (n = 482) areas were surveyed. A 27-item questionnaire was used to collect data about sources that patients searched for medical information, patient awareness and use of Cochrane systematic reviews and other EBM resources, and their demographic characteristics.

**Results:**

Half of the patients searched for medical information from sources other than physician. Internet was the most common place they searched for information. Very few patients indicated using EBM sources for medical information; one fifth of patients heard of EBM and 4% of the patients heard of the Cochrane Collaboration. Patients considered physician’s opinion as the most reliable source of medical information. A logistic regression model showed that educational level and urban *vs* rural residence were the predictors of awareness about EBM and systematic reviews (*P* < 0.001 for both).

**Conclusion:**

Our finding that patients consider a physician’s opinion to be the most reliable source of health-related information could be used for promotion of high-quality health information among patients. More effort should be devoted to the education of patients in rural areas and those with less formal education. New avenues for knowledge translation and dissemination of high-quality health information among patients are necessary.

Healthcare service provides advice, examinations, interventions, surgery, and any other form of medical treatment that is legally approved and in accordance with medical methods and principles (1). Considering that the aim of medical care is to benefit patients, then patients are the best persons who can assess the utility of health service (2). A “person-centered care” is defined as “providing care that is respectful of and responsive to individual patient preferences, needs, and values, and ensuring that patient values guide all clinical decisions” (3). This represents a shift from the paternalistic approach of health care professionals to partnership (4). Although this ideal is rarely accomplished, patients have the right to understand their illness, treatment options, and prognosis regardless of whether they decide to participate in clinical decisions (5,6).

With the increasing availability of the Internet, more and more patients search for available information, which requires that such information be accurate, unbiased, and understandable (7). A survey of 1002 consumers found that 57% of respondents turn to the Internet for information that answers their personal medical questions (8). Furthermore, international studies showed that 72% of Internet users in the USA and 71% in Europe conduct health-related searches (9,10). This shows that there is a shift in the role of patient from a passive recipient to an active consumer of health information.

Growing awareness for synthesizing numerous evidence that is used in medicine has led to the development of evidence-based medicine (EBM), the purpose of which is to integrate the best available clinical evidence, clinical expertise, and patient values, needs, and desires (11).

EBM is characterized by the synthesis of primary studies into systematic reviews. There are hundreds of thousands systematic reviews available today (12), but the current gold standard in EBM are Cochrane systematic reviews (13). To make Cochrane evidence available to patients, each Cochrane systematic review contains a plain language summary (PLS) written in short format and simple lay language.

In 2008, Cochrane Croatia was founded at the School of Medicine in Split, Croatia (14), with the main goal to promote EBM and systematic reviews among medical students, health care providers, and general population (15). One of the most popular activities of Cochrane Croatia is translation of PLSs to Croatian language. These PLSs are freely available to the public online and via social media (16).

Considering increasing availability of Cochrane evidence on interventions and diagnostic accuracy in Croatian language, it would be useful to assess the effect of the Cochrane Croatia knowledge translation and dissemination activities among patients. Having conducted a national survey on the awareness and knowledge of EBM among physicians (17), this study aimed to determine the use of EBM information and awareness and knowledge of EBM among patients.

## METHODS

### Sample

In this cross-sectional study, we aimed to include a sample of 1000 general practice (GP) patients from Croatia. From each of the 5 Croatian regions (central Croatia, northwestern Croatia, eastern Croatia, north Adriatic and Lika, middle and south Adriatic), one GP practice was selected from urban and one from rural part of the region. Urban GP practices were located in the cities of Čakovec, Osijek, Rijeka, Split, and Zagreb. Rural GP practices were located in Kotoriba, Kupinečki Kraljevec, Donji Muć, Viškovo, and Vuka.

### Participants

The aim was to include 100 patients per GP practice in each region. The participants received instructions to fill out the questionnaire in the waiting room, in a private area. The participants were invited consecutively until there were 50 respondents who were women and 50 who were men, to ensure gender balance. If a patient refused to participate, the recruitment process continued on to the next patient until the target number of 100 participants was reached.

The inclusion criteria were ≥18 years of age. Exclusion criteria were underage, cognitive disorders, and mental health disorders that could preclude proper understanding of the questionnaire.

After reading the written information about the study, patients signed a written informed consent and completed an anonymous questionnaire about the awareness and use of EBM information sources.

### Questionnaire

For the purpose of this study, we developed a 27-item questionnaire with open-ended and multiple-choice questions. The questionnaire was piloted among 10 researchers affiliated with Cochrane Croatia, 10 students (who were not exposed to EBM principles), and 10 lay persons to ensure that the wording was appropriate and that the questionnaire takes into consideration appropriateness of both the content and the language. During this, we kept in mind that the questionnaire would be self-administered by lay persons and particular attention was paid to developing plain language. Feedback from the pilot testing was incorporated (changes made on the questions 1, 2, 5, and 25) and once again the questionnaire was pilot-tested on these individuals to ensure content-related, criterion-related, and construct validity of the questionnaire.

Patients were asked about the frequency of visiting physicians, searching for additional information about their therapy and/or diagnosis, Internet use, using medical information sources, their knowledge of EBM, systematic reviews, The Cochrane Library, Cochrane Croatia, and if they were reading Cochrane PLSs translated to Croatian. Their demographic data were also collected, including professional status, education level, sex, and age. Patients also evaluated the reliability of medical information sources and commented on their experience in case they discussed the medical information they found on their own with their physician. English translation of the questionnaire is provided as supplementary material (Supplementary questionnaire) (supplementary questionnaire). The survey was conducted between September 2014 and September 2015.

### Statistical analysis

Each questionnaire received a unique code and every answer was coded with numerical indicators. Data were entered into a piloted data extraction sheet. Descriptive data analysis was performed. Data were expressed as frequencies with percentages or as medians with interquartile ranges (IQR) because the data were not normally distributed, as shown by Kolmogorov-Smirnov test. Comparison of various categorical variables for possible differences between rural and urban areas or regions was conducted using χ^2^ test. EBM or systematic review awareness with residence, sex, education level, employment status, visits to physician, and search for health information from sources other than a physician were also tested with χ^2^ test. The relation between awareness of EBM or systematic reviews and age was tested using Mann-Whitney test. Logistic regression was used to determine the contribution of several variables to EBM or systematic review awareness. Analyses were conducted with MedCalc statistical software, v 15.2.1 (© MedCalc Software bvba, Ostend, Belgium). The level of statistical significance was set at *P* < 0.05.

## RESULTS

A total of 978 patients were included in the study. Their median age was 47 years (IQR, 34-59 years). Most patients were employed and had high school-level education. Patients in urban and rural areas had very similar employment and education status in all five geographic regions (Table 1). The majority of patients in both urban and rural areas visited a physician 3-4 times a year, but in two regions the majority of patients indicated visiting physician 1-2 times a year (Table 1).

**Table 1:** General characteristics of study population

Variable	No. (%) of participants							
	Entire sample (N = 978)	Rural areas (n = 482)	Urban areas (n = 496)	Osijek region (n = 189)	Rijeka region (n = 193)	Split region (n = 200)	Varaždin region (n = 199)	Zagreb region (n = 197)
Age (years, median, interquartile range)	47 (34-59)	44 (33-56)	48 (35-62)	45 (35-54)	51 (35-67)	48 (36-62.25)	41 (29-52)	50 (35-62)
Sex (total)	969	479	490	189	193	197	198	192
men	481 (49.6)	235 (49.1)	246 (50.2)	92 (48.7)	96 (49.7)	98 (49.7)	100 (50.5)	95 (49.5)
women	488 (50.4)	244 (50.9)	244 (49.8)	97 (51.3)	97 (50.3)	99 (50.3)	98 (49.5)	97 (50.5)
Employment status (total)	967	479*	488*	187	193*	195	197*	195
employed	489 (50.6)	258 (53.9)	231 (47.3)	108 (57.7)	75 (38.9)	95 (48.7)	114 (57.9)	97 (49.7)
unemployed	160 (16.5)	95 (19.8)	65 (13.3)	40 (21.4)	32 (16.6)	29 (14.9)	34 (17.3)	25 (12.8)
student	66 (6.8)	18 (3.8)	48 (9.9)	5 (2.7)	12 (6.2)	13 (6.7)	22 (11.1)	14 (7.2)
retired	252 (26.1)	108 (22.5)	144 (29.5)	34 (18.2)	74 (38.3)	58 (29.7)	27 (13.7)	59 (30.3)
Education level (total)	966	478*	488*	187	193	195	198	193
primary or lower	131 (13.6)	89 (18.6)	42 (8.6)	25 (13.4)	25 (13.0)	27 (13.8)	32 (16.2)	22 (11.4)
secondary	603 (62.4)	296 (61.9)	307 (62.9)	126 (67.4)	112 (58.0)	123 (63.1)	124 (62.6)	118 (61.1)
college/university	216 (22.4)	85 (17.8)	131 (26.9)	32 (17.1)	51 (26.4)	44 (22.6)	41 (20.7)	48 (24.9)
Master or PhD	16 (1.6)	8 (1.7)	8 (1.6)	4 (2.1)	5 (2.6)	1 (0.5)	1 (0.5)	5 (2.6)
Frequency of visiting a physician/year								
never	29	20	9	12	8	4	4	1
1-2 times	271	144	127	56	57	47	56	55
3-4 times	311	159	152	46	55	75	68	67
6-10 times	205	94	111	39	32	46	39	49
>10 times	156	64	92	36	40	25	30	25

*χ^2^ test (*P* < 0.05).

Half of the patients indicated that they engage in search for medical information on diagnosis or therapy in other places, ie, they do not only ask their physician. Their most common source of medical information was the Internet, followed by ‘friends and acquaintances who are health care workers’. As many as 77% of the patients used Internet, with almost identical percentage of Internet use in urban and rural areas (79% *vs* 76%, respectively; Table 2). Among 739 patients who indicated using Internet when searching for medical information, there were 658 (89%) who searched for medical information on Internet search engines, such as Google, while a very few patients indicated using PubMed or EBM databases (Table 2).

**Table 2:** Participants’ search for medical information

Variables	No. (%) of participants							
	Entire sample (N = 978)	Rural areas (n = 482)	Urban areas (n = 496)	Osijek region (n = 189)	Rijeka region (n = 193)	Split region (n = 200)	Varaždin region (n = 199)	Zagreb region (n = 197)
Searching for information about diagnosis or therapy other than with physician?	954	473*	481*	185	188	198	197	186
yes	491 (51.5)	224 (47.4)	267 (55.5)	98 (53.0)	92 (49.0)	111 (56.1)	90 (45.7)	100 (53.8)
no	463 (48.5)	249 (52.6)	241 (44.5)	87 (47.0)	96 (51.0)	87 (43.9)	107 (54.3)	86 (46.2)
Sources of medical information^†^	776	366	410	132	165	161	179	139
books	181 (23.3)	77 (21.0)	104 (25.4)	31 (23.5)	42 (25.5)	20 (12.4)	47 (26.3)	41 (29.5)
friends and acquaintances that are health care workers	343 (44.2)	162 (44.3)	181 (44.1)	62 (47.0)	65 (39.4)	92 (57.1)	71 (39.7)	53 (38.1)
research manuscripts	97 (12.5)	39 (10.7)	58 (14.1)	17 (12.9)	25 (15.2)	7 (4.3)	25 (14.0)	23 (16.5)
promotional materials of pharmaceutical companies	90 (11.6)	42 (11.5)	48 (11.7)	5 (3.8)	29 (17.6)	14 (8.7)	19 (10.6)	23 (16.5)
Internet	552 (71.1)	253 (69.1)	299 (72.9)	93 (70.5)	101 (61.2)	117 (72.7)	127 (70.9)	114 (82.0)
specialized magazines about medicine for patients	105 (13.5)	38 (10.4)	67 (16.3)	8 (6.1)	32 (19.4)	16 (9.9)	9 (5.0)	40 (28.8)
other	41 (5.3)	20 (5.5)	21 (5.1)	3 (2.3)	21 (12.7)	14 (8.7)	3 (1.7)	0 (0.0)
Do you use Internet?	958	465*	493*	189	191	197	197*	184
never	219 (22.9)	99 (21.3)	120 (24.3)	46 (24.3)	58 (30.4)	41 (20.8)	36 (18.3)	38 (20.7)
yes, few times a month	94 (9.8)	59 (12.7)	35 (7.1)	21 (11.1)	24 (12.6)	15 (7.6)	20 (10.1)	14 (7.6)
yes, few times a week	187 (19.5)	108 (23.2)	79 (16.0)	48 (25.4)	34 (17.8)	41 (20.8)	24 (12.2)	40 (21.7)
yes, daily	458 (47.8)	199 (42.8)	259 (52.5)	74 (39.2)	75 (39.2)	100 (50.8)	117 (59.4)	92 (50.0)
If you are using Internet for searching information about the medicine, which Internet information source do you use?^†^	739	366	373	143	133	156	161	146
Internet search engines (Google, etc.)	658 (89.0)	340 (92.9)	318 (85.3)	121 (84.6)	119 (89.5)	131 (84.0)	146 (90.7)	141 (96.6)
PubMed	23 (3.1)	7 (1.9)	16 (4.3)	4 (2.8)	2 (1.5)	6 (3.8)	7 (4.3)	4 (2.7)
specialized evidence-based medicine databases	37 (5.0)	19 (5.2)	18 (4.8)	7 (4.9)	8 (6.0)	8 (5.1)	12 (7.5)	2 (1.4)
other	20 (2.7)	5 (1.4)	15 (4.0)	2 (1.4)	6 (4.5)	1 (0.6)	6 (3.7)	5 (3.4)

*χ^2^ test (*P* < 0.05)

†Multiple choice question.

About one-fifth (19%) of patients indicated they had heard of EBM (Table 3). More patients in urban than rural areas answered positively to this question (25% vs 13%, respectively). The percentages of patients who reported to have heard of systematic reviews, The Cochrane Collaboration, a Cochrane systematic review, Cochrane summary or Cochrane translation were low. Among a few patients who indicated they heard of Cochrane, majority indicated that they came across it on PubMed or other medical database or the Cochrane summaries website. There were 3% of patients who indicated that they heard of The Cochrane Library, mostly from friends and acquaintances who are health care workers, from a research paper, and the Internet. Of 25 patients who indicated they had heard of The Cochrane Library, 13 indicated that they used it and mostly accessed it from a computer at work. The Cochrane Library was used rarely and most participants did found it useful (Table 3).

**Table 3:** Use of evidence-based medicine information sources

Variables	No. (%) of participants							
	Entire sample (N = 978)	Rural areas (n = 482)	Urban areas (n = 496)	Osijek region (n = 189)	Rijeka region (n = 193)	Split region (n = 200)	Varaždin region (n = 199)	Zagreb region (n = 197)
Did you ever hear about evidence-based medicine?	956	473*	483*	189*	185	192	194	196
yes	183 (19.1)	60 (12.7)	123 (25.5)	59 (31.2)	32 (17.3)	25 (13.0)	39 (20.1)	28 (14.2)
no	773 (80.6)	413 (87.3)	360 (74.5)	130 (68.8)	153 (82.7)	167 (87.0)	155 (79.9)	168 (85.8)
Did you ever hear about a type of research called a “systematic review”?	960	475	485	188	187	197	192*	196
yes	167 (17.4)	91 (19.2)	76 (15.7)	29 (15.4)	31 (16.6)	33 (16.8)	48 (25.0)	26 (13.3)
no	793 (82.6)	384 (80.8)	409 (84.3)	159 (84.6)	156 (83.4)	164 (83.2)	144 (75.0)	170 (86.7)
Did you ever hear about The Cochrane Collaboration?	966	477	489	189	191	197	194	195
yes	42 (4.3)	17 (3.6)	25 (5.1)	6 (3.2)	8 (4.2)	8 (4.1)	12 (6.2)	8 (4.1)
no	924 (95.7)	460 (96.4)	464 (94.9)	183 (96.8)	183 (95.8)	189 (95.9)	182 (93.8)	187 (95.9)
Did you ever hear about Cochrane systematic review or a Cochrane summary, a Cochrane translation?	949	470	479	188	184	196	187	194
yes	27 (2.9)	10 (2.1)	17 (3.5)	2 (1.0)	4 (2.2)	7 (3.6)	10 (5.3)	4 (2.1)
no	922 (97.1)	460 (97.9)	462 (96.5)	186 (99.0)	180 (97.8)	189 (96.4)	177 (94.7)	190 (97.9)
If yes, where did you hear about it?^†^	25	8	17	2	3	7	9	4
The Cochrane Library	4 (16.0)	1 (12.5)	3 (17.6)	0 (0.0)	0 (0.0)	2 (28.6)	2 (22.2)	0 (0.0)
web site with Cochrane summaries	9 (36.0)	1 (12.5)	8 (47.1)	1 (50.0)	0 (0.0)	3 (42.9)	2 (22.2)	3 (75.0)
PubMed or other medical database	14 (56.0)	4 (50.0)	10 (58.8)	2 (100.0)	0 (0.0)	5 (71.4)	7 (77.8)	0 (0.0)
Internet portal about health	4 (16.0)	3 (37.5)	1 (5.9)	0 (0.0)	2 (66.7)	1 (14.3)	1 (11.1)	0 (0.0)
Facebook	4 (16.0)	1 (12.5)	3 (17.6)	1 (50.0)	0 (0.0)	1 (14.3)	2 (22.2)	0 (0.0)
other social network	1 (4.0)	0 (0.0)	1 (5.9)	0 (0.0)	0 (0.0)	0 (0.0)	0 (0.0)	1 (25.0)
other	1 (4.0)	0 (0.0)	1 (5.9)	0 (0.0)	1 (33.3)	0 (0.0)	0 (0.0)	0 (0.0)
Did you ever hear about The Cochrane Library?	945	465	480	188	188	191	186	192
yes	24 (2.5)	8 (1.7)	16 (3.3)	2 (1.1)	3 (1.6)	7 (3.7)	8 (4.3)	4 (2.1)
no	921 (97.5)	457 (98.3)	464 (96.7)	186 (98.9)	185 (98.4)	184 (96.3)	178 (95.7)	188 (97.9)
Where did you hear about The Cochrane Library? ^†^	23	7	16	2	2	7	8	4
books	4 (17.4)	1 (14.3)	3 (18.6)	0 (0.0)	0 (0.0)	3 (42.9)	1 (12.5)	0 (0.0)
friends and acquaintances that are health care workers	11 (47.8)	3 (42.9)	8 (50.0)	1 (50.0)	0 (0.0)	5 (71.4)	1 (12.5)	4 (100.0)
research manuscripts	10 (43.5)	2 (28.6)	8 (50.0)	0 (0.0)	1 (50.0)	5 (71.4)	3 (37.5)	1 (25.0)
promotional materials of pharmaceutical companies	2 (8.7)	0 (0.0)	2 (12.5)	0 (0.0)	0 (0.0)	1 (14.3)	1 (12.5)	0 (0.0)
Internet	9 (39.1)	3 (42.9)	6 (37.5)	1 (50.0)	2 (100.0)	3 (42.9)	3 (37.5)	0 (0.0)
specialized magazines about medicine for patients	1 (4.3)	0 (0.0)	1 (6.3)	0 (0.0)	0 (0.0)	1 (14.3)	0 (0.0)	0 (0.0)
other	3 (13.0)	1 (14.3)	2 (12.5)	0 (0.0)	0 (0.0)	1 (14.3)	2 (25.0)	0 (0.0)
Do you ever use Cochrane Library?	25	9	16	2	3	7	9	4
yes	13 (52.0)	5 (55.6)	8 (50.0)	2 (100.0)	0 (0.0)	5 (71.4)	6 (66.7)	0 (0.0)
no	12 (48.0)	4 (44.4)	8 (50.0)	0 (0.0)	3 (100.0)	2 (28.6)	3 (33.3)	4 (100.0)
How do you access Cochrane Library? ^†^	12	4	8	2	0	5	5	0
work computer	6 (50.0)	2 (50.0)	4 (50.0)	2 (100.0)	0 (0.0)	2 (40.0)	2 (40.0)	0 (0.0)
home computer	10 (83.3)	2 (50.0)	8 (100.0)	0 (0.0)	0 (0.0)	5 (100.0)	5 (100.0)	0 (0.0)
mobile phone	0 (0.0)	0 (0.0)	0 (0.0)	0 (0.0)	0 (0.0)	0 (0.0)	0 (0.0)	0 (0.0)
other	0 (0.0)	0 (0.0)	0 (0.0)	0 (0.0)	0 (0.0)	0 (0.0)	0 (0.0)	0 (0.0)
How often do you use Cochrane Library?	12	4	8	2	0	5	5	0
less than once a year	1 (8.3)	0 (0.0)	1 (12.5)	0 (0.0)	0 (0.0)	1 (20.0)	0 (0.0)	0 (0.0)
once a year	3 (25.0)	1 (25.0)	2 (25.0)	0 (0.0)	0 (0.0)	1 (20.0)	2 (40.0)	0 (0.0)
once in 6 months	3 (25.0)	2 (50.0)	1 (12.5)	1 (50.0)	0 (0.0)	2 (40.0)	0 (0.0)	0 (0.0)
once a week	4 (33.4)	1 (25.0)	3 (37.5)	1 (50.0)	0 (0.0)	1 (20.0)	2 (40.0)	0 (0.0)
more times a week	1 (8.3)	0 (0.0)	1 (12.5)	0 (0.0)	0 (0.0)	0 (0.0)	1 (20.0)	0 (0.0)
How useful did you find information from Cochrane Library?	11	3	8	2	0	5	4	0
useless	1 (9.1)	0 (0.0)	1 (12.5)	0 (0.0)	0 (0.0)	1 (20.0)	0 (0.0)	0 (0.0)
undecided	0 (0.0)	0 (0.0)	0 (0.0)	0 (0.0)	0 (0.0)	0 (0.0)	0 (0.0)	0 (0.0)
useful	10 (90.9)	3 (100.0)	7 (87.5)	2 (100.0)	0 (0.0)	4 (80.0)	4 (100.0)	0 (0.0)

*χ^2^ test (*P* < 0.05).

^†^Multiple choice question.

In our sample, there was a significant difference between patients from urban and rural areas in the number of patients-students, patients with primary-level education, and university-level education (χ^2^ test, *P* < 0.001 for all), whether they searched for information on diagnosis or therapy other than with physician (χ^2^ test, *P* = 0.012), and how often they used the Internet (χ^2^ test, *P* < 0.001). When different regions from our sample were compared, there was a significant difference between the number of employed patients (the lowest number in the Rijeka region; χ^2^ test, *P* < 0.001), number of retired patients (the lowest number in Varaždin region; χ^2^ test, *P* < 0.001), and in daily use of Internet (the highest number in Varaždin region; χ^2^ test, *P* < 0.001) (Tables 1 and 2).

Higher awareness of EBM was associated with the urban place of residence (χ^2^ test, *P* < 0.001), higher level of education (χ^2^ test, *P* < 0.001), and employment status (χ^2^ test, *P* = 0.001).

Patients from Osijek region were most familiar with EBM compared with other regions (χ^2^ test, *P* < 0.001). Awareness about systematic reviews was associated with higher level of education (χ^2^ test, *P* < 0.001) and employment status (χ^2^ test, *P* < 0.001). In comparison with other regions, patients from Varaždin region were most familiar with systematic review term (χ^2^ test, *P* = 0.002).

Among 181 patients who heard about EBM, there were only 61 (34%) patients who heard about both EBM and systematic review. Among 160 participants who heard about a systematic review, 99 (61.9%) did not hear about EBM (χ^2^ test, *P* < 0.001). Using the logistic regression model, we showed that the educational level and urban/rural residence were predictors for awareness about EBM and awareness about systematic reviews (*P* < 0.001 for both).

When asked to describe in their own words the meaning of EBM, 129 participants responded (Supplementary Table 1) (supplementary Table 1). Majority (n = 102) answered that it was medicine based on research, verified medicine, tested on a large number of patients. The second most common group of answers indicated that EBM is a Western, or European, or non-alternative, recognized medicine (n = 5). Many patients equated EBM with the use of certain medical therapies, and one patient answered that EBM is “the TV show of Dr. Oz”.

Only 3% of patients heard of the Cochrane Croatia and 2% visited ‘Cochrane Health’ Facebook page. When asked if they read translated Cochrane PLSs, 2% of patients gave a confirmatory answer. When ranking usefulness of various information sources, physician’s opinion was ranked the highest, followed by systematic reviews, research conducted on humans and experiences of other patients, while information from media were ranked the lowest (Table 4).

**Table 4:** Participants’ awareness of Cochrane Croatia activities

Variables	No. (%) of participants							
	Entire sample (N = 978)	Rural areas (n = 482)	Urban areas (n = 496)	Osijek region (n = 189)	Rijeka region (n = 193)	Split region (n = 200)	Varaždin region (n = 199)	Zagreb region (n = 197)
Did you ever hear about Cochrane Croatia?	927	462	465	186	180	190	180	191
yes	31 (3.3)	11 (2.4)	20 (4.3)	4 (2.2)	3 (1.7)	8 (4.2)	10 (5.6)	6 (3.1)
no	896 (96.7)	451 (97.6)	445 (95.7)	182 (97.8)	177 (98.3)	182 (95.8)	170 (94.4)	185 (96.9)
Did you ever visit a ‘Cochrane health’ page on Facebook?	928	463	465	187	183	187	178	193
yes	15 (1.6)	5 (1.1)	10 (2.2)	2 (1.1)	3 (1.6)	3 (1.6)	5 (2.8)	2 (1.0)
no	913 (98.4)	458 (98.9)	455 (97.8)	185 (98.9)	180 (98.4)	184 (98.4)	173 (97.2)	191 (99.0)
Did you ever read translated plain language summaries in Croatian language prepared by the Cochrane Croatia?	934	468	466	187	182	188	183	194
yes	22 (2.4)	7 (1.5)	15 (3.2)	4 (2.1)	3 (1.6)	5 (2.7)	7 (3.8)	3 (1.5)
no	912 (97.6)	461 (98.5)	451 (96.8)	183 (97.9)	179 (98.4)	183 (97.3)	176 (96.2)	191 (98.5)
Please rate these medical information sources based on your perception of their reliability (median, interquartile range)								
experiences of other patients	3 (2-4)	3 (3-4)	3 (2-4)	2 (2-3)	3 (2-4)	3 (3-4)	3 (2-3)	4 (3-4)
research conducted on humans	4 (3-5)	4 (3-5)	4 (3-4)	3 (3-4)	4 (3-4)	5 (4-5)	4 (3-4)	4 (3-5)
analysis of multiple studies (a systematic review)	4 (3-5)	4 (4-5)	4 (3-5)	3 (3-4)	4 (3-5)	5 (4-5)	4 (3-4)	4 (4-5)
information from media	3 (2-3)	3 (2-4)	2 (2-3)	2 (1-3)	3 (2-4)	3 (2-3)	3 (2-3)	2 (2-3)
physician’s opinion	5 (4-5)	5 (4-5)	5 (4-5)	5 (5-5)	5 (4-5)	5 (4-5)	4 (4-5)	5 (4-5)

When asked whether they ever discuss medical information they found on their own with their physician, 245 patients confirmed it. Majority said that the physician’s reaction was positive or good, and only a few patients reported a negative reaction from a physician (Supplementary Table 2) (supplementary Table 2).

## DISCUSSION

We found that half of the patients searched for medical information from sources other than physician and mostly on the Internet. Very few patients used EBM sources of medical information; one-fifth of patients heard of the EBM, but very few heard of the Cochrane. Patients considered the physician’s opinion as the most reliable source of medical information. Place of residence and educational level were predictors of awareness about EBM and systematic reviews.

Health information seeking is crucial for today’s participatory role of patients in health care (18). Information seeking is noted as a critical component of shared decision-making because informed patients will more likely participate in choosing their care, make wiser decisions, and adhere more fully to the treatment (19). This study addressed general health information seeking habits, with a special focus on the Cochrane evidence as the gold standard in EBM information. Since Cochrane Croatia was founded, a significant volunteer effort was devoted to translating PLSs into Croatian language and their promotion via social media (20). Our results indicate that additional strategies need to be considered to reach a wider consumer audience in order to disseminate Cochrane evidence, but keeping focus on dissemination through web and social media is also relevant.

Although social media are relatively new, they are already used in health care. There are reports about different patterns of social media use between patients and health care professionals, indicating that patients prefer using Facebook for social support and exchanging advice (21). Web has a huge potential as a resource for high-quality public health information. However, since there are various forms of information, this can lead to unintended consequences, such as misinformation and patient fraud (22). Therefore, it is important to promote high-quality, non-commercial EBM information sources among patients.

Cochrane Reviews have considerably expanded their reach and the access to the Cochrane Database of Systematic Reviews, where the reviews are published, has been increasing continuously (23). Since Cochrane Reviews are prepared and published in English, the Cochrane Collaboration has devoted a considerable effort to translating the evidence for use by patients, ie, the public. Different strategies could be used for targeted dissemination to specific patient groups (24). Translating summaries of Cochrane into multiple languages is an important part of the Cochrane strategy titled “Evidence to everyone, everywhere” (23). At the moment, Croatian is one of thirteen languages to which Cochrane PLSs are being translated.

In this study, most respondents defined EBM as a practice of medicine justified by research results. It was curious that one person wrote that EBM was the Dr Oz Show. Contents of this TV show were recently scrutinized by researchers. Their research concluded that consumers should be very skeptical about recommendations provided on medical talk shows, as only a third to one-half of the recommendations given was based on believable or somewhat believable evidence (25).

When asked to rank different sources of medical information, patients indicated physician’s opinion as the most reliable, followed by a systematic review, as an analysis of multiple studies. Information from the media was ranked the lowest. According to the EBM principles, systematic reviews are the highest level in the hierarchy of evidence in medicine (26), whereas expert opinion is ranked much lower, even lower than observational studies (27). However, that does not mean that clinical expertise is irrelevant in EBM. On the contrary, the practice of EBM involves integrating the appraisal of evidence with clinical expertise and patient values to apply the results in clinical practice (26). Our findings that patients consider physician’s opinion as the most reliable source of medical information could be used for knowledge translation and physicians need to be enticed to promote EBM information sources, such as The Cochrane Library and translated Cochrane evidence, among patients. Curricula of medical specialty training for physicians then also need to include EBM and knowledge translation skills, if they are not already included. Since we found that the predictors of awareness about EBM and systematic reviews were the place of residence and educational level, more effort should be devoted to education of patients in rural areas and those with less education.

A limitation of this study may be patient sampling, which was convenient and, therefore, can be considered non-representative of five large geographic areas. However, we made an effort to include patients from both urban and rural areas, from as much as 10 different family medicine practices. The sample was not entirely balanced, as in rural areas more participants had lower educational level. Second, the sample was drawn from patients visiting a physician. Third, it is possible that participants gave positive answers to questions about EBM and systematic review awareness even though they were not familiar with these concepts. Still, very few patients responded positively to those questions and responses were uniform between regions. Other limitations may be related to authors’ professional activities, as several study authors are personally involved in the activities of Cochrane Croatia and have a track record of publications on EBM. However, only one of them participated in data collection, without giving any instructions about the manuscript to the recruited participants. To ensure that we were not biased when creating our questionnaire, it has been piloted and validated among researchers, students, and lay persons. Finally, there is potentially an upward bias present in our results.

While a number of studies about knowledge, awareness, and attitudes about EBM were conducted among various types of health care workers in different settings worldwide (17,28-31), we believe this is the first study that investigates awareness and use of EBM information among patients on a nation-wide scale, which is a strength of this study.

Even though half of the included patients used Internet in their search for medical information, very few were familiar with EBM, sources such as the Cochrane and few used these sources. Place of residence and education level were predictors of awareness about EBM and systematic reviews. Our finding that patients find physician’s opinion to be the most reliable source of health-related information could be used for promotion of high-quality health information among patients and an incentive to train physicians in EBM and knowledge translation skills.

## Additional Material

Supplementary questionnaire
