# Supplementary material for: Awareness and use of evidence-based medicine information among patients in Croatia: a nation-wide cross-sectional study
Source: Croat Med J. 2017 Aug;58(4):300–1. doi: 10.3325/cmj.2017.58.300 (PMC5577645; doi:10.3325/cmj.2017.58.300)
Supplement: Supplementary questionnaire [file CroatMedJ_58_s001.pdf]

# QUESTIONNAIRE

1. How many times a year do you visit a physician(s) (family physician and/or other physicians)?

□ Never □ 1-2 times □ 3-4 times □ 6-10 times □ more than 10 times

2. Did you ever search for health information about your diagnosis or therapy in places other than a physician?

□ Yes □ No

3. Where do you search for health information? (multiple answers allowed)

□ Books
□ Friends and acquaintances that are healthcare workers
□ Research manuscripts
□ Promotional materials of pharmaceutical companies
□ Internet
□ Specialized magazines about medicine for patients
□ Other (please indicate): ……………………………………………………………………………………………

4. Do you use Internet (at home, work or elsewhere)?

□ Never □ Few times a month □ Few times a week □ Daily

5. If you are using Internet for searching information about the medicine, which Internet information source do you use?

□ Internet search engines (such as Google, etc.)
□ PubMed
□ Specialized databases for EBM
□ Other (please indicate):……………………………………………………………………………………………

6. Did you ever hear about "evidence-based medicine"?

□ Yes □ No

*If the answer is „No", please go to the question 8.*

7. Please describe in your own words what is the meaning of "evidence-based medicine"?

……………………………………………………………………………………………………………………………
……………………………………………………………………………………………………………………………
……………………………………………………………………………………………………………………………

8. Did you ever hear about a type of research called a "systematic review"?

□ Yes □ No

9. Did you ever hear about The Cochrane Collaboration and its activity?

□ Yes □ No

10. Did you ever hear about Cochrane systematic review (or a Cochrane summary, a Cochrane translation,...)?

□ Yes □ No

*If the answer is „No", please go to the question 12.*

11. If "Yes", where did you hear about it?

□ The Cochrane Library
□ Web site with Cochrane summaries
□ PubMed or other medical database
□ Internet portal about health
□ Facebook
□ Other social network
□ Other (please indicate): ……………………………………………………………………………………………

12. Did you ever hear about The Cochrane Library?

□ Yes □ No

*If the answer is „No", please go to the question 18.*

13. **Where did you hear about The Cochrane Library? (multiple answers allowed)**

□ Books
□ Friends and acquaintances that are healthcare workers
□ Research manuscripts
□ Promotional materials of pharmaceutical companies
□ Internet
□ Specialized magazines about medicine for patients
□ Other (please indicate): …………………………………………………………………………………………………………………………

14. **Do you ever use The Cochrane Library?**

□ Yes □ No

*If the answer is „No", please go to the question 18.*

15. **How do you access The Cochrane Library (multiple answers allowed)?**

□ Work computer □ Home computer □ Mobile phone □ Other

16. **How often do you use The Cochrane Library?**

□ Less than once a year
□ Once a year
□ Once in 6 months
□ Once a week
□ More times a week

17. **How useful did you find information from The Cochrane Library?**

□ Useless □ Undecided □ Useful

18. **Did you ever hear about Cochrane Croatia?**

□ Yes □ No

19. **Did you ever visit a 'Cochrane health' page on Facebook?**

□ Yes □ No

20. **Did you ever read translated plain language summaries in Croatian language prepared by the Cochrane Croatia?**

□ Yes □ No

21. **Please rate these medical information sources based on your perception of their reliability. (1= completely unreliable, 5= completely reliable). Select your choice.**

| | | | | | |
|---|---|---|---|---|---|
| Experiences of other patients | 1 | 2 | 3 | 4 | 5 |
| Research conducted on humans | 1 | 2 | 3 | 4 | 5 |
| Analysis of multiple studies (a systematic review) | 1 | 2 | 3 | 4 | 5 |
| Information from media | 1 | 2 | 3 | 4 | 5 |
| Physician's opinion | 1 | 2 | 3 | 4 | 5 |

22. **Did you ever discuss with your physician (family physician and/or other physicians) medical information that you have found?**

□ Yes □ No

23. **What were the physicians' reactions if you talked about the medical information that you have found?**

……………………………………………………………………………………………………………………………………………………………………
……………………………………………………………………………………………………………………………………………………………………

24. **Employment status:**

□ Employed
□ Unemployed
□ Student
□ Retired

25. **Your education:**

□ Primary school or less
□ Secondary school
□ College/university
□ Master or PhD

26. **Sex**

□ Men □ Women

27. **Age**

………… years
