# Supplementary material for: Awareness and use of evidence-based medicine information among patients in Croatia: a nation-wide cross-sectional study
Source: Croat Med J. 2017 Aug;58(4):300–1. doi: 10.3325/cmj.2017.58.300 (PMC5577645; doi:10.3325/cmj.2017.58.300)
Supplement: Supplementary Table 1 [file CroatMedJ_58_s002.pdf]

Supplementary table 1. Patients' definitions of evidence-based medicine

**Answer**	**No. of patients**
Based on research/science; verified; tested on a large number of patients	102
Western medicine, European medicine, not alternative, official medicine	5
Based on experience of people that were treated with certain methods and drugs	2
Healing	2
Treatments administered after examination, blood draws, tests and established diagnosis	2
Treatment with drugs and therapies, surgery	1
Existence of information about diseases, causes, consequences and treatment	1
Safe treatment	1
Precise information	1
Help for further treatment	1
Accurate source of information	1
Medicine based on some previous cases	1
Treatment based on results from the practice	1
Tested treatment that was approved by insurance fund	1
Improvement of life	1
Medicine based on some achievements from certain authors	1
Advancement of medicine, which is contributing to a progress of citizens, and solving some problems related to diseases	1
The most reliable type of medicine	1
Any treatment that can be explained with arguments	1
Therapy for diabetes, therapy for high blood pressure	1
The TV show of Dr. Oz	1
