# Supplementary material for: Awareness and use of evidence-based medicine information among patients in Croatia: a nation-wide cross-sectional study
Source: Croat Med J. 2017 Aug;58(4):300–1. doi: 10.3325/cmj.2017.58.300 (PMC5577645; doi:10.3325/cmj.2017.58.300)
Supplement: Supplementary Table 2 [file CroatMedJ_58_s003.pdf]

Supplementary table 2. Physicians' reactions after hearing that patients search for medical information elsewhere

**Phyician's reaction**	**No. (%) of patients (n=245)**
Great/enthusiastic	14 (5.7)
Positive/good	195 (79.6)
Neutral	17 (6.9)
Not recommended	8 (3.3)
Bad/negative/overreacting	11 (4.5)
